# Prognostic Factors for Mortality Following Diaphragmatic Herniorrhaphy in Dogs and Cats: Multivariable Logistic Regression and Machine Learning Approaches

**DOI:** 10.3390/vetsci12090819

**Published:** 2025-08-26

**Authors:** Irin Kwananocha, Sirirat Niyom, Pharkpoom Budsayaplakorn, Suwicha Kasemsuwan, Wutthiwong Theerapan, Kanawee Warrit

**Affiliations:** 1 Center for Veterinary Research and Innovation, Faculty of Veterinary Medicine, Kasetsart University, Bangkok 10900, Thailand; irin.kwananocha@gmail.com; 2 Department of Companion Animal Clinical Sciences, Faculty of Veterinary Medicine, Kasetsart University, Bangkok 10900, Thailand; wutthiwong.t@ku.th; 3 Kasetsart University Veterinary Teaching Hospital, Faculty of Veterinary Medicine, Kasetsart University, Bangkok 10900, Thailand; pharkpoom.b@ku.th; 4 Department of Veterinary Public Health, Faculty of Veterinary Medicine, Kasetsart University, Bangkok 10900, Thailand; suwicha.k@ku.th; 5 Faculty of Veterinary Medicine, Chiang Mai University, Chiang Mai 50100, Thailand; kanawee.w@cmu.ac.th

**Keywords:** cat, diaphragmatic hernia, dog, machine learning, mortality, prognosis, random forest

## Abstract

**Simple Summary:**

Traumatic diaphragmatic hernia is a serious injury for dogs and cats that can cause respiratory compromise due to a ruptured diaphragm. This study investigated factors associated with the risk of death in dogs and cats undergoing surgery for this condition. Both traditional statistical analysis and a machine learning technique known as random forest were used to analyze the data. The findings showed that animals with chronic hernias (ruptured for over 14 days) and those with elevated blood urea nitrogen had a higher risk of mortality. Overall survival was approximately 79%, with slightly higher rates observed in dogs (82.5%) than in cats (77%). The random forest algorithm produced results consistent with traditional methods, suggesting it may hold promise for future applications in medical data analysis.

**Abstract:**

This study aimed to explore prognostic factors for mortality in dogs and cats following traumatic diaphragmatic herniorrhaphy using both multivariable logistic regression, a traditional statistical method, and the random forest algorithm, a machine learning approach. Associations between demographic and clinical variables and mortality were examined. Overall survival was 78.8% (149/189), 77% (97/126) in cats and 82.5% (52/63) in dogs. Key findings revealed that chronic diaphragmatic hernia (DH) significantly increased the odds of death compared to acute cases (adjusted odds ratio (OR) = 4.01, 95% confidence interval (CI): 1.69–9.53). Elevated blood urea nitrogen (BUN) increased mortality (adjusted OR = 3.24, 95% CI: 1.22–8.57). Cox proportional hazards analysis revealed that chronic DH (adjusted hazard ratio (HR) = 3.31, 95% CI: 1.51–7.30) and elevated BUN (HR = 2.88, 95% CI: 1.23–6.77) were associated with increased one-year mortality risk. The random forest analysis reinforced these findings, identifying hernia duration (Gini importance: 1.90) and BUN (Gini importance: 0.94) as the most crucial predictors. Among chronic DH patients, 55% of those with elevated BUN experienced fatal outcomes based on classification and regression tree (CART) analysis. The consistency of random forest results with logistic regression strengthens the reliability of these prognostic insights for DH patients.

## 1. Introduction

Over the last two decades, survival rates following surgical treatment for traumatic diaphragmatic hernia (DH) in dogs and cats have been reported to range from 73.3% to 93.3% [1,2,3,4,5,6,7,8]. Despite efforts to identify risk factors associated with mortality, definitive evidence regarding the influence of surgical timing or hernia duration on mortality in affected animals remains elusive [9]. Furthermore, the primary risk factor has shown inconsistency across studies. For instance, Legallet (2017) [4] reported an increase in mortality associated with concurrent injuries and prolonged operation duration in 79 dogs and 17 cats, while patient characteristics such as age, sex, and neuter status did not emerge as significant factors [4]. In contrast, studies focusing on feline patients revealed that age was significantly associated with mortality: older cats were at greater risk of mortality following diaphragmatic herniorrhaphy compared to younger cats [6,10].

A 1987 study suggested that the duration of hernia could influence mortality risk in canine patients, with notably higher mortality rates observed in those subjected to surgical repair within 24 h post-trauma or more than a year afterward [11]. However, subsequent research involving 63 dogs and 29 cats refuted the significance of early surgical intervention in perioperative mortality rates [2]. These discrepancies may be attributed to variations in the clinical variables examined, differences in sample sizes, disparities in disease severity and management, and differences in the proportions of dogs and cats across studies. Additionally, variations in the timing of case collection may significantly impact outcomes and the identification of risk factors, as changes in surgical techniques and medical approaches over time could influence the results [1,2,3,4,5,6,7,8,9,10,11,12].

Given the potential variability in risk factors associated with DH mortality across species, differences in management approaches, and periods of case collection, the aim of this study was to meticulously collect data from DH animals treated at a high-caseload referral veterinary teaching hospital over a short, defined period. The first objective was to determine the mortality rate of DH patients post-herniorrhaphy and identify prognostic factors using traditional statistical methods such as multivariable logistic regression.

The second objective was to apply a machine learning technique, specifically the random forest algorithm, to compare its results with those of traditional statistical methods. Machine learning methods are capable of uncovering complex, nonlinear relationships between clinical variables and outcomes that may not be easily detected by conventional statistical approaches [13]. Through this approach, the study aimed to assess whether machine learning could identify additional, more intricate prognostic factors, providing a deeper understanding of the factors influencing survival outcomes in DH patients.

It was hypothesized that factors such as hernia duration, physical status, age, preoperative biochemical parameters, clinical characteristics, and perioperative factors would be associated with postoperative mortality in DH patients. Furthermore, the random forest algorithm was expected to identify prognostic variables comparable to those detected by multivariable logistic regression.

The random forest algorithm has gained traction in clinical decision support systems, particularly for prognosis prediction [13,14,15,16,17,18,19], due to its ability to manage complex medical datasets, handle missing values, and mitigate overfitting issues. During training, multiple decision trees are created, each contributing either a classification result by voting on the most common class or a regression result by averaging predictions. Each tree is built from a random subset of both training data and features, which helps reduce overfitting and enhances generalization performance. The trees split the data based on feature values, selecting the optimal split point at each node until they reach a stopping criterion, such as a predefined maximum depth or insufficient samples. By using a random subset of features at each node, random forest reduces the correlation among trees and further prevents overfitting. The final prediction aggregates results from all trees, employing either majority voting for classification or averaging for regression, creating a robust model that captures intricate data relationships without succumbing to overfitting. Additionally, the random forest algorithm allows clinicians to understand the relative importance of different features, aiding in identifying key variables that drive predictions across various classification tasks [20,21].

## 2. Materials and Methods

The study was approved by the Kasetsart University Institutional Animal Care and Use Committee under protocol ACKU67-VET-052. All medical records of DH patients that underwent surgical intervention at Kasetsart University Veterinary Teaching Hospital in Thailand between January 2018 and March 2023 were reviewed. The hospital is a referral facility that consistently manages approximately 20 to 30 DH cases annually, drawing patients from diverse regions across the country. Information obtained from the medical records included species, age, sex, weight, preoperative blood profiles, hernia duration, American Society of Anesthesiologists (ASA) physical status, timing of surgical correction post-trauma, anesthetic drugs used, anesthesia period, number of surgical procedures performed during the same anesthesia period, herniated organs, concurrent injuries (soft tissue, orthopedic, and neurological injuries), and coexisting diseases. Patients who survived until hospital discharge were categorized as survivors, while those who did not survive until hospital discharge were classified as deceased. Telephone interviews were conducted with owners to gather information on long-term outcomes one year post-surgery.

Acute DH was defined as a hernia duration of 14 days or less at the time of surgery, while chronic DH was defined as a hernia duration of more than 14 days. Hernia duration was defined as the time interval from the reported or documented traumatic event to the time of surgery, based on owner-provided history and/or medical records. This definition reflects the estimated time since trauma rather than the time from diagnosis. Blood profiles falling within the reference range (Table A1) were considered normal, while those outside the range were deemed abnormal. Patients were also classified into two groups based on their age (<5 years vs. ≥5 years), ASA status (<4 vs. ≥4), number of surgical procedures (1 procedure vs. >1 procedure), weight of the cat (<3 kg vs. ≥3 kg), and weight of the dog (<10 kg vs. ≥10 kg).

The classification into two groups for each variable to facilitate statistical analysis was based on the previous literature and clinical relevance. The age cut-off of 5 years was selected according to evidence that dogs aged ≥5 years have an increased risk of mortality related to general anesthesia or sedation, with those aged 5–7 years having 4.9 times the odds of anesthesia-related death compared to those aged 0.5–1.5 years [22]. Similarly, ASA status was dichotomized (<4 vs. ≥4) to reflect the substantial increase in anesthesia-related mortality, with dogs having ASA scores of 4–5 showing 19.0 times the odds compared to those with ASA 1–2 [22]. The number of procedures performed during the same anesthetic period was categorized as a single versus more than one procedure, as multiple procedures may prolong anesthesia, increase surgical stress, and result in greater cumulative tissue injury, all of which have been associated with higher complication and mortality rates. Body weight cut-offs (<3 kg for cats and <10 kg for dogs) were determined based on the authors’ clinical experience in anesthetic practice at a referral hospital, where patients below these thresholds were often more difficult to maintain normothermic. Lower body weight predisposes animals to perioperative hypothermia due to a higher surface area-to-volume ratio and has been associated with increased odds of anesthetic-related death [23].

Statistical analysis was primarily conducted using Number Cruncher Statistical System (NCSS) statistical software (version 21.0.2; NCSS, LLC, Kaysville, UT, USA). Histograms, skewness, kurtosis, and Shapiro-Wilk tests were used to assess the normality of continuous variables. Descriptive statistics such as means and standard deviations are reported for normally distributed variables, while medians and ranges are presented for variables not normally distributed. Categorical variables are expressed as frequencies and percentages. Univariable logistic regression analysis was employed to examine the associations between mortality and patient characteristics and clinical information obtained from medical records. Variables with a *p* value < 0.2 in the univariable analysis were included in forward stepwise selection with logistic regression models. The chi-squared test was utilized to evaluate the relationship between two categorical factors, with only relevant factors—defined as those meeting the *p*-value threshold (<0.2) in the univariable analysis, prioritized by the smallest p-values, and having biological plausibility and/or support from previous literature—being incorporated into the multivariable logistic regression analysis. Model discrimination was assessed by calculating the area under the receiver operating characteristic (ROC) curve (AUC). The model fit was examined by calculating the log likelihood and pseudo R^2^.

Random forest modeling was conducted using the “randomForest” R package (version 4.7-1.1) in R software (version 4.3.2; R Foundation for Statistical Computing, Vienna, Austria). The random forest imputation function was used to handle missing data, with features having no more than 20% missing data imputed. Features (variables) with more than 20% missing data were excluded from the analysis. The ranks of the Gini importance of each variable were determined from the decrease in Gini impurity. The random forest classifier was optimized for the number of variables tested at each split (mtry) and the number of trees (ntree) using repeated (n = 100) tenfold cross-validation with the “caret” R package (version 6.0-94). The performance metric used for parameter selection was classification accuracy averaged across the resampling iterations. This cross-validation approach allowed for a robust estimation of model performance while minimizing the risk of overfitting, ensuring that the selected parameters would generalize well to unseen data. Classification and regression tree (CART) analysis, utilizing the two most significant factors determined by the random forest model, was conducted using the “rpart” R package (version 4.1.23). The random forest algorithm was performed twice: (1) using all variables and (2) excluding highly correlated variables (*p* < 0.001), which were tested by Pearson’s chi-squared test.

The Kaplan-Meier test was conducted to demonstrate and visualize one-year survival between dogs and cats. To provide supplementary comparison with the primary analyses, a multivariable Cox proportional hazard regression analysis was performed to compare the findings from the logistic regression and random forest models with a time-to-event approach. Observations were considered censored if the patient survived until the end of the one-year follow-up period, was lost to follow-up, or died from a cause unrelated to the disease. Variables identified as significant in the final multivariable logistic regression model and the top three Gini importance factors were entered directly into the Cox regression model as covariates. Hazard ratios (HRs) with 95% confidence intervals (CIs) were calculated to evaluate the association between each variable and survival time. The proportional hazard assumption was assessed for each variable using Schoenfeld residuals. Variables violating the assumption were considered for stratification or excluded from the final model. A significance level of 0.05 was used for all the statistical tests.

## 3. Results

In sum, 189 patients with DH, consisting of 126 cats and 63 dogs, were included in the study. Twelve patients were excluded from the survival analysis due to loss to follow-up or death from other causes. The median age was 7 months (range 1 month to 12 years) for cats and 3 years (range 2 months to 18.3 years) for dogs. Of the total, 33 dogs (52.4%) and 57 cats (45.2%) were male. The weights of the DH cats ranged from 0.47 to 6.1 kg, with a median of 2.6 kg, while those of the DH dogs ranged from 1.5 to 33.5 kg, with a median of 8 kg. In cats, car accidents were the most common cause of DH, accounting for 52.4% (66/126), followed by bites (4.8%, 6/126) and falls (2.4%, 3/126). Unknown causes constituted 40.5% (51/126) of the cases. In dogs, car accidents accounted for 66.7% (42/63) of DH cases, followed by bites (11.1%, 7/63) and hits inflicted by a person (1.6%, 1/63). Unknown causes represented 20.6% (13/63) of the cases.

The majority of the cats were domestic shorthair cats (92.1%, 116/126), followed by Persian cats (4.8%, 6/126), British shorthairs (1.6%, 2/126), Bengals (0.8%, 1/126), and Scottish folds (0.8%, 1/126). Among dogs, mixed-breed dogs constituted the largest proportion (63.5%, 40/63), followed by poodles (9.5%, 6/63), Chihuahuas (4.8%, 3/63), golden retrievers (4.8%, 3/63), shih tzus (4.8%, 3/63), Pomeranians (3.2%, 2/63), and other breeds such as Bangkeaws, French bulldogs, Jack Russells, Labrador retrievers, miniature pinschers, and spitz, each accounting for 1.6% (1/63).

Coexisting diseases in the patients included heart disease (7.9%, 3/38), blood parasite infection (15.8%, 6/38), mammary gland tumor (2.6%, 1/38), splenic mass (2.6%, 1/38), renal disease (21.1%, 8/38), megacolon (2.6%, 1/38), urogenital disease (18.4%, 7/38), feline infectious peritonitis (2.6%, 1/38), feline immunodeficiency virus (7.9%, 3/38), feline leukemia virus (13.2%, 5/38), and convulsion (5.3%, 2/38). Concurrent surgeries performed in the patients included abdominal wall suturing (24.1%, 7/29), wound reconstruction (24.1%, 7/29), thoracic wall suturing (20.7%, 6/29), ovariohysterectomy (17.2%, 5/29), splenectomy (10.3%, 3/29), intestinal resection and anastomosis (6.9%, 2/29), prepubic urethrostomy (6.9%, 2/29), tail amputation (3.4%, 1/29), and nephrectomy (3.4%, 1/29). Some patients underwent more than one procedure during the same anesthetic period. The variable “anesthesia period” was excluded from analysis, as data for this variable were missing in 58 of 189 records (30.7%).

The overall survival rate, calculated across both species, was 78.8% (149/189). Among the cats, 97 out of 126 (77%) survived, while 52 out of 63 dogs (82.5%) remained alive following surgical intervention. Table 1 presents the demographic and clinical characteristics, as well as comparisons between patients who survived and those who died after diaphragmatic herniorrhaphy. The duration of hernia, leukocytosis, BUN concentrations, and presence of intestine in the thoracic cavity (herniated intestine) differed between the two groups of patients (*p* = 0.002, *p* = 0.049, *p* = 0.026, and *p* = 0.006, respectively) (Table 1).

### 3.1. Logistic Regression

According to the univariable logistic regression analysis for mortality, the duration of hernia, BUN, and the presence of herniated intestine were significant prognostic factors (*p* = 0.003, *p* = 0.030, and *p* = 0.008, respectively), while age, leukocytosis, alanine aminotransferase (ALT) concentration, and coexisting disease were borderline significant prognostic factors (*p* = 0.161, *p* = 0.051, *p* = 0.112, and *p* = 0.192, respectively) (Table 2).

The correlations between potential prognostic factors from the univariable analysis were examined using the chi-squared test. The results indicated associations between the duration of hernia and herniated intestine (*p* < 0.001), duration of hernia and leukocytosis (*p* < 0.001), duration of hernia and ALT (*p* < 0.001), and coexisting disease and age (*p* < 0.001). Therefore, leukocytosis, ALT, herniated intestine, and coexisting disease were not considered for inclusion in the multivariable logistic regression analysis.

**Figure 1:**
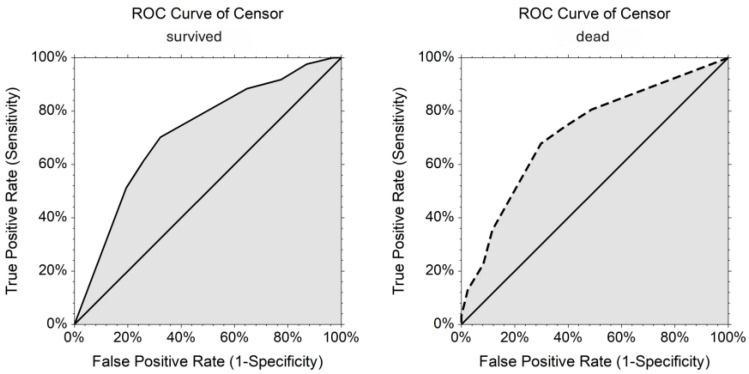
Receiver operating characteristic (ROC) curves for the final logistic regression model predicting mortality in diaphragmatic hernia (DH) patients. The model achieved an area under the curve (AUC) of 0.72 (95% CI: 0.61–0.83) for survivors and 0.72 (95% CI: 0.63–0.81) for non-survivors. The solid line corresponds to the ROC curve for survivors and the dashed line corresponds to the ROC curve for non-survivors, matching the panel titles.

The final logistic regression model is presented in Table 3. After adjusting for other covariates, mortality was greater in chronic DH animals (adjusted odds ratio (OR) = 4.01, 95% CI: 1.69, 9.53), DH patients with elevated BUN (adjusted OR = 3.24, 95% CI: 1.22, 8.57), and animals aged ≥5 years (adjusted OR = 1.91, 95% CI: 0.76, 4.85). The log likelihood was −68.66, and the model R^2^ was 0.24. The model demonstrated moderate performance, with an AUC of 0.720 (95% CI: 0.61, 0.83) for surviving patients and 0.720 (95% CI: 0.63, 0.81) for non-surviving patients (Figure 1).

### 3.2. Random Forest

**Figure 2:**
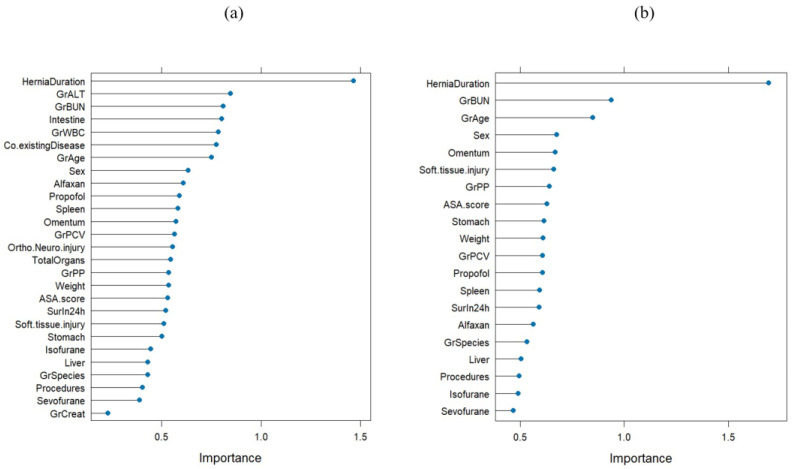
The top 20 features for predicting postoperative mortality, as ranked by Gini importance from random forest analysis with the inclusion of all variables (**a**) and the exclusion of highly correlated features (**b**). The x-axis represents the importance value derived from the random forest analysis.

The Gini importance rank, calculated from the decrease in Gini impurity using the random forest algorithm, is illustrated in Figure 2. The algorithm was performed twice—(1) with all variables included and (2) with highly correlated variables excluded (*p* < 0.001)—as shown in Figure 2a,b, respectively. In the model including all variables, the three most influential predictors were hernia duration, ALT, and BUN, with Gini importance values of 1.46, 0.85, and 0.81, respectively, and an out-of-bag (OOB) error rate of 22.2%. After removing highly correlated variables, the top three predictors were hernia duration, BUN, and age, with Gini importance values of 1.90, 0.94, and 0.85, respectively, and an OOB error rate of 21.05%. These findings indicate that hernia duration consistently ranked as the most important predictor, regardless of variable correlation structure.

**Figure 3:**
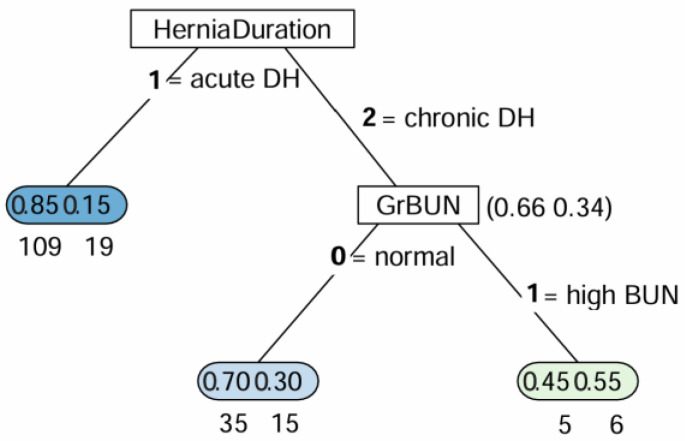
Decision tree model for mortality of patients after diaphragmatic herniorrhaphy. Each box represents the percentage of patients with distinguishing variables identified through classification and regression tree (CART) analysis. Fractional numbers inside the boxes indicate patients who died (on the right side) and those who survived (on the left side).

The decision tree-based classification for mortality in animal patients after diaphragmatic herniorrhaphy is depicted in Figure 3. The duration of hernia was identified as the variable for the initial split. Among patients diagnosed with chronic DH, 34% died and 66% survived. For the second separation among chronic DH patients, BUN was the determining variable: 55% of patients with elevated BUN experienced fatal outcomes, while 45% survived.

### 3.3. Survival Analysis

**Figure 4:**
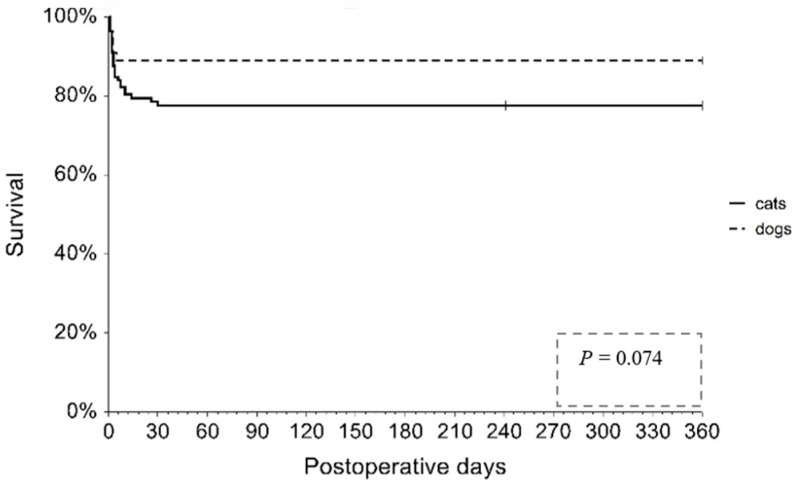
Kaplan–Meier survival curves of dogs and cats with diaphragmatic hernia over one year.

Figure 4 displays the results of the Kaplan-Meier survival analysis to delineate differences in the one-year survival rate among DH patients categorized by species. There was no significant difference in survival rates between dogs and cats (83% and 77%, respectively; *p* = 0.074). Notably, there was no substantial increase in the mortality rate observed after the third day post-surgery in dogs or after the thirtieth day post-surgery in cats. Specifically, medical records indicated only two feline mortalities occurring more than two weeks post-surgery: one on day 30 and another on day 26. Both cats were classified as chronic DH patients. Imaging diagnoses for these cases revealed a hiatal hernia with gastroesophageal intussusception obstruction in the first cat and megaesophagus that developed after herniorrhaphy in the second. Unfortunately, despite scheduled appointments for surgical correction, both cats passed away before the procedures could be carried out.

In the multivariable Cox proportional hazard regression using the variables retained from the final multivariable logistic regression model and the top three Gini importance factors identified hernia duration and BUN category as significant predictors of one-year survival. Cats and dogs with chronic hernia had a 3.31-fold higher hazard of death compared with those with acute hernia (HR = 3.31, 95% CI: 1.51, 7.30; *p* = 0.003). Elevated BUN was also significantly associated with reduced survival (HR = 2.88, 95% CI: 1.23, 6.77; *p* = 0.015). Age was not significantly associated with survival (HR = 1.49, 95% CI: 0.63, 3.55; *p* = 0.367) (Table 4). A likelihood ratio test indicated that the overall Cox regression model was statistically significant (χ^2^ = 12.87, *p* = 0.005), suggesting that the covariates as a group were a good fit for the data. The proportional hazard assumption was assessed using the Schoenfeld residual test. The assumption was found to be valid for all variables in the model (hernia duration: *p* = 0.81; age: *p* = 0.17; BUN: *p* = 0.63). The global test also showed no evidence of a violation of the assumption (*p* = 0.51).

## 4. Discussion

The survival rate following diaphragmatic herniorrhaphy in both dogs and cats in this study was 78.8%, consistent with reported survival rates ranging from 73.3% to 93.3% over the last two decades [1,2,3,4,5,6,7,8]. The investigation revealed that the duration of hernia and preoperative BUN concentrations were associated with mortality outcomes. In particular, the mortality rate for patients diagnosed with chronic DH was 34.4% (21 out of 61), while for those with acute DH, it was 14.8% (19 out of 128). These findings align with postoperative mortality rates delineated in two studies from Turkey, which indicated higher mortality rates in feline subjects with chronic DH compared to their acute DH counterparts, specifically 19% vs. 16.1% [3] and 30.8% vs. 16.7% [24], respectively. In contrast, these results diverge from prior investigations by Gibson (2005) [2] and Legallet (2017) [4], which found no association between hernia duration and mortality. One possible explanation for this discrepancy is the proportions of dogs and cats across studies, as the current study included a larger sample of cats (126 cats and 63 dogs), in line with the two reports from Turkey that focused solely on DH cats [3,24], whereas the studies in 2005 and 2017 had a greater proportion of dog patients (79 dogs and 17 cats and 63 dogs and 29 cats, respectively) [2,4]. This is supported by the findings from the comparison analysis between survivors and deceased patients (Table 1), which highlighted a significant impact of hernia duration on feline patient mortality (*p* = 0.017) and a less pronounced association in dogs (*p* = 0.052). However, additional factors contributing to the disparities across studies may include variations in patient characteristics, perioperative and postoperative management, and other unaccounted variables.

The higher mortality rate in chronic DH may be due to the fact that surgical treatment for chronic DH is typically more complex than for acute DH [25]. Over time, the tissue surrounding the hernia undergoes pathological changes, making the repositioning of displaced organs more challenging. The hernia may be harder to reduce, and adhesions within the thorax can impede the process. Vascular control is crucial during adhesion division, and some organs, such as the omentum or lung lobes, may need to be resected if they are compromised. Necrosis and reherniation are potential complications that must be closely monitored after hernia closure to prevent postoperative issues [1]. Additionally, reperfusion injury following the reduction of chronically displaced organs may contribute to increased mortality and other negative outcomes in these patients [11,25]. Overall, the complexity of the procedure increases with chronic cases, requiring more extensive preparation and careful management, particularly in smaller animals like cats, which, as seen in this study and previous research [3,24], tend to have a higher mortality rate.

In this study, an increase in BUN was associated with a higher mortality rate, while an increase in creatinine did not exhibit a similar association. Furthermore, among individuals with chronic DH, the mortality rate reached 55% in those with elevated BUN. Generally, serum urea concentrations rise in scenarios where renal clearance is compromised, such as in cases of renal failure or impairment [26]. However, elevated urea concentrations can also occur in conditions unrelated to renal disorders, such as upper gastrointestinal bleeding, dehydration, catabolic states (e.g., infection and fever), and consumption of a high-protein diet [26,27]. Additionally, BUN also serves as a valuable marker for neurohormonal activity, as dysfunction in both cardiac and renal systems, along with disturbances in neurohormonal balance, can lead to elevated BUN concentrations [28,29]. These elevated concentrations have been associated with higher mortality in various human diseases. Several studies have shown a strong association between elevated BUN concentrations and mortality in patients with conditions such as heart failure [30,31,32,33,34], acute pancreatitis [35], and COVID-19 pneumonia [36]. In critically ill human patients, elevated BUN concentrations at admission have been found to be independently associated with increased mortality [37,38], with one study specifically demonstrating this association even in patients with normal serum creatinine concentrations [37]. Therefore, consistent with prior evidence, BUN concentrations show promise as a significant prognostic factor for mortality in animal patients following diaphragmatic herniorrhaphy.

The age of DH patients was found to be a factor of borderline significance in terms of mortality (*p* = 0.171). Although statistical significance was not evident after adjusting for covariates in the multivariable analysis, it is crucial to interpret these findings with caution. In particular, these results appear to differ from those of previous studies conducted on cats, which suggested that younger cats had a greater likelihood of survival than their older counterparts [6,9]. The inconsistency could be attributed to the limited representation of elderly patients in this study, which included only 10 cats aged over 5 years compared to 116 younger patients. In addition, data from dogs included in this study may have influenced the results.

The presence of the intestine in the thoracic cavity appeared to impact mortality risk among DH patients, as suggested by the univariable analysis. However, this factor was excluded from the multivariable analysis due to its strong correlation with hernia duration. Herniated intestine was predominantly observed in chronic DH patients, which was associated with fourfold the risk of mortality compared to acute DH. The univariable analysis also indicated that leukocytosis and elevated ALT levels might be associated with reduced mortality. These associations could be due to the strong correlation between leukocytosis and elevated ALT levels with acute DH, which has a lower mortality. Leukocytosis is likely a common inflammatory response to acute injury [39], while elevated ALT levels may indicate liver damage following trauma [40].

Performing surgery within 24 h of trauma did not adversely affect patient survival. This result is consistent with a retrospective study conducted in Ontario (2005) [2], but contradicts an earlier report by Boudrieau and Muir (1987) [11], which noted a significantly higher mortality rate when surgery was performed within 24 h after trauma. This disparity may be attributed to potential advancements in patient management over time, particularly in handling emergency and/or traumatized patients.

This study revealed no discernible associations between mortality and concurrent soft tissue, orthopedic, or neurological injuries. These findings contrast with previous investigations that indicated a heightened risk of death among DH patients with concurrent injuries [4,6,9]. However, significant correlations were observed between hernia duration and the presence of concurrent injuries. Acute DH cases frequently present with multiple injuries, likely due to the force causing diaphragmatic rupture, which may also damage other tissues. In contrast, chronic DH patients are typically presented to the hospital after their initial injuries have healed. The lack of a direct association between concurrent injuries and mortality in this study might be influenced by the complex intercorrelations among factors within real-world clinical data, making definitive conclusions challenging with the current sample size. To clarify these relationships, future studies with larger samples are needed, particularly focusing on acute DH patients both with and without concurrent injuries. Similarly, coexisting diseases were excluded from the multivariable analysis due to their substantial correlation with patient age, as older patients tend to have a higher likelihood of having coexisting conditions. If coexisting diseases are considered an important prognostic factor, future studies should investigate this in more detail.

The field of medicine has recently witnessed the integration of advanced technologies for evaluating and predicting the likelihood of incidents and the expected outcomes of various diseases. Recent evidence indicates that machine learning algorithms have advantages over traditional prediction models because they can handle large, complex, and diverse datasets more effectively [41]. Among these options, the random forest algorithm has become well known for its ability to combine the outputs of several decision trees and produce a single valid outcome. The widespread use of this technology is fueled by its intuitive interface and adaptable capabilities in addressing both classification and regression problems, making it a promising tool for generating prognostic assumptions and medical decisions [16,17,18,21].

The random forest algorithm uses the mean decrease in Gini impurity to assess the importance of variables/features, presenting them in order of significance. Gini impurity measures the probability of a variable being incorrectly classified at a node for each feature [17]. A greater mean decrease in Gini impurity indicates greater importance of a feature in achieving accurate predictions, as it leads to a substantial reduction in Gini impurity across all splits of all trees within the random forest. In this study, the algorithm provided results consistent with those of traditional statistical analysis. The duration of hernia had the greatest Gini importance, followed by BUN concentrations, after excluding highly correlated features. Therefore, the duration of hernia was crucial for predicting the mortality of DH patients following herniorrhaphy and significantly influenced the model’s decision-making process. This finding completely agrees with results from multivariable logistic regression and aligns with the Cox proportional hazard model, which indicated that animals with chronic DH had a poorer survival rate than those with acute DH. Furthermore, the decision tree-based classification provided additional insights: specifically, when BUN was the second split, the mortality rate of chronic DH patients with elevated BUN levels increased further, as illustrated in Figure 2.

The contrasting results from the random forest models—one using all features and the other excluding highly correlated ones—underscore the importance of the preprocessing step to eliminate such features and highlight the need for careful interpretation in medical research when models contain correlated features. While a feature might not directly associate with the outcome, it could correlate with other impactful predictors. Although random forest models are theoretically capable of accurate prediction even with collinearities [17]—given that the impurity removed by the first correlated feature is not revisited by subsequent ones, potentially lessening the latter’s apparent importance—this study demonstrated that failing to remove correlated features in preprocessing can still affect the outcome and impact the algorithm’s interpretability. Despite these challenges, both model configurations (with all features and with correlated features excluded) were still able to accurately pinpoint the most influential factors, and their results closely aligned with traditional statistical methods.

Various methodologies have been proposed in recent years to address the challenge of correlated features, including group-selection algorithms, feature clustering, permutation importance, and modern techniques such as recursive feature elimination, supervised and unsupervised feature clustering, knockoff variable importance metrics, and mutual forest impact and mutual impurity reduction [42,43,44,45,46]. However, a consensus on the most efficient approach is currently lacking. In this study, the selection or exclusion of highly correlated variables was based on background scientific knowledge and their significant impact on the outcome, as determined by the univariable analysis. While variable grouping algorithms or other contemporary techniques were not employed, the outcomes remained consistent when compared to those of the multivariable analysis. Nonetheless, it is important to note that the dataset in this study was relatively small and full of background information. For larger datasets or scenarios with limited background knowledge, modern approaches may prove more suitable.

Several inherent limitations of this study must be acknowledged. First, due to its retrospective nature, the data were extracted from routine medical records, which were not originally standardized or formatted for specific research objectives. This introduces potential for inconsistencies in data collection and missing information for variables not routinely documented. Consequently, certain crucial variables previously identified as potential influences on DH patient mortality (e.g., perioperative oxygen dependence), were not consistently recorded or available for analysis. Furthermore, reliance on client-provided history carries an inherent risk of recall bias, particularly for subtle details pertaining to injury onset or the date of death. Secondly, although this study included a relatively large number of patients compared to similar investigations conducted in the last two decades, its single-center origin limits the generalizability of the findings. Clinical practices, referral patterns, available resources, and the specific patient population demographics can vary significantly between institutions, thereby introducing institutional bias. Therefore, the insights gained from this study warrant validation through future prospective, multicenter studies incorporating external data, which would substantially strengthen the external validity and generalizability of the results across diverse clinical settings.

In addition, several methodological limitations should be noted. The primary aim was to identify prognostic factors rather than to optimize predictive performance; therefore, a comprehensive set of performance metrics and advanced interpretability analyses were not performed in this study. Variable importance was assessed using Gini impurity, although permutation importance could provide complementary insights in larger datasets. Partial-dependence or accumulated local effect plots for the top predictors were not generated. These could further clarify the nature of predictor–outcome relationships in future work. Continuous predictors were dichotomized for clinical interpretability and direct comparison with the logistic regression model, which may obscure nonlinear relationships and reduce statistical power. Lastly, although stratified cross-validation was used to minimize bias from the moderate class imbalance, more advanced resampling or weighting techniques may be required to address imbalance in larger datasets.

## 5. Conclusions

This study identified hernia duration as the primary prognostic factor for mortality in DH patients, with preoperative elevated BUN concentrations also significantly associated with increased mortality. These findings, consistently observed across both traditional multivariable analysis and random forest machine learning, underscore the poorer survival rates in small animal patients with chronic DH (ruptured for over 14 days). The random forest algorithm proved valuable in this study, accurately predicting mortality and identifying the most significant prognostic factors, aligning well with traditional statistical methods and Cox regression analysis. However, the study also highlighted that preprocessing to remove highly correlated features is crucial for improved interpretability and outcome accuracy in clinical applications.

These crucial findings offer valuable insights for informing pretreatment clinical decision-making in patients diagnosed with diaphragmatic rupture. Implementing tailored management strategies, such as invasive stabilization and assigning dedicated surgical teams for high-risk patients, could potentially improve outcomes.

## Figures and Tables

**Table 1:** Characteristics of surviving and deceased patients following diaphragmatic herniorrhaphy.

Variables		Dogs (n = 63)			Cats (n = 126)			Both Dogs and Cats (N = 189)		
		Survival n (%)	Death n (%)	*p* Value	Survival	Death	*p* Value	Survival	Death	*p* Value
Sex	Female	23 (76.7%)	7 (23.3%)	0.325	52 (75.4%)	17 (24.6%)	0.634	75 (75.8%)	24 (24.2%)	0.277
	Male	29 (87.9%)	4 (12.1%)		45 (79.0%)	12 (21.0%)		74 (82.2%)	16 (17.8%)	
Age	<5 years	36 (90.0%)	4 (10.0%)	0.081 ^a^	90 (77.6%)	26 (22.4%)	0.695 ^a^	126 (80.8%)	30 (19.2%)	0.157
	≥5 years	16 (69.6%)	7 (30.4%)		7 (70.0%)	3 (30.0%)		23 (69.7%)	10 (30.3%)	
Weight (kg)	Dog <10/cat <3	33 (86.8%)	5 (13.2%)	0.267	62 (76.5%)	19 (23.5%)	0.875	95 (79.8%)	24 (20.2%)	0.662
	Dog ≥10/cat ≥3	19 (76.0%)	6 (24.0%)		35 (77.8%)	10 (22.2%)		54 (77.1%)	16 (22.8%)	
Duration of hernia	Acute	39 (88.6%)	5 (11.4%)	0.052	70 (83.3%)	14 (16.7%)	0.017*	109 (85.2%)	19 (14.8%)	0.002 *
	Chronic	13 (68.4%)	6 (31.6%)		27 (64.3%)	15 (35.7%)		40 (65.6%)	21 (34.4%)	
Blood profiles										
PCV	Normal	32 (84.2%)	6 (15.8%)	0.667	62 (72.9%)	23 (27.1%)	0.121	94 (76.4%)	29 (23.6%)	0.268
	Anemia	20 (80.0%)	5 (20.0%)		35 (85.4%)	6 (14.6%)		55 (83.3%)	11 (16.7%)	
WBC	Normal	20 (87.0%)	3 (13.0%)	0.732 ^a^	47 (68.1%)	22 (31.9%)	0.009 *	67 (72.8%)	25 (27.2%)	0.049 *
	Leukocytosis	32 (80.0%)	8 (20.0%)		50 (87.7%)	7 (12.3%)		82 (84.5%)	15 (15.5%)	
Plasma protein	Normal	29 (87.9%)	4 (12.1%)	0.197 ^a^	62 (76.5%)	19 (23.5%)	0.718	91 (79.8%)	23 (20.2%)	0.686
	Abnormal	20 (74.1%)	7 (25.9%)		31 (79.5%)	8 (20.5%)		51 (77.3%)	15 (22.7%)	
BUN	Normal	39 (83.0%)	8 (17.0%)	0.387 ^a^	64 (83.1%)	13 (16.9%)	0.039 *	103 (83.1%)	21 (16.9%)	0.026 *
	Elevated	7 (70.0)	3 (30.0%)		11 (61.1%)	7 (38.9%)		18 (64.3%)	10 (35.7%)	
Creatinine	Normal	43 (82.7%)	9 (17.3%)	0.631 ^a^	80 (78.4%)	22 (21.6%)	1.000 ^a^	123 (79.9%)	31 (20.1%)	0.712 ^a^
	Elevated	6 (75.0%)	2 (25.0%)		3 (75.0%)	1 (25.0%)		9 (75.0%)	3 (25.0%)	
ALT	Normal	11 (78.6%)	3 (21.4%)	0.698 ^a^	18 (66.7%)	9 (33.3%)	0.096	29 (70.7%)	12 (29.3%)	0.107
	Elevated	35 (83.3%)	7 (16.7%)		64 (82.0%)	14 (18.0%)		99 (82.5%)	21 (17.5%)	
Anesthetics	Propofol	46 (83.6%)	9 (16.4%)	0.620 ^a^	88 (77.9%)	25 (22.1%)	0.494 ^a^	134 (79.8%)	34 (20.2%)	0.378
	Alfaxalone	6 (75.0%)	2 (25.0%)	0.620 ^a^	8 (66.7%)	4 (33.3%)	0.469 ^a^	14 (70.0%)	6 (30.0%)	0.306
	Isoflurane	23 (85.2%)	4 (14.8%)	0.745 ^a^	48 (76.2%)	15 (23.8%)	0.832	71 (78.9%)	19 (21.1%)	0.987
	Sevoflurane	29 (80.6%)	7 (19.4%)	0.745 ^a^	49 (77.8%)	14 (22.2%)	0.832	78 (78.8%)	21 (21.2%)	0.987
Surgical procedure	1 procedure	42 (85.7%)	7 (14.3%)	0.243 ^a^	83 (74.8%)	28 (25.2%)	0.188 ^a^	125 (78.1%)	35 (21.9%)	0.574
	>1 procedure	10 (71.4%)	4 (28.6%)		14 (93.3%)	1 (6.7%)		24 (82.8%	5 (17.2%)	
Herniated organ										
Stomach	no	29 (87.9%)	4 (12.1%)	0.325 ^a^	51 (73.9%)	18 (26.1%)	0.440	80 (78.4%)	22 (21.3%)	0.959
	yes	23 (76.7%)	7 (23.3%)		40 (80.0%)	10 (20.0%)		63 (78.8%)	17 (21.3%)	
Liver	no	16 (76.2%)	5 (23.8%)	0.348	23 (85.2%)	4 (14.8%)	0.307 ^a^	39 (81.3%)	9 (18.8%)	0.629
	yes	36 (85.7%)	6 (14.3%)		70 (74.5%)	24 (25.5%)		106 (77.9%)	30 (22.1%)	
Spleen	no	34 (85.0%)	6 (15.0%)	0.498	63 (74.1%)	22 (25.9%)	0.272	97 (77.6%)	28 (22.4%)	0.561
	yes	18 (78.3%)	5 (21.7%)		30 (83.3%)	6 (16.7)		48 (81.4%)	11 (18.6%)	
Intestine	no	27 (90.0%)	3 (10.0%)	0.189 ^a^	38 (88.4%)	5 (11.6%)	0.026 *	65 (89.0%)	8 (11.0%)	0.006 *
	yes	25 (75.8%)	8 (24.2%)		55 (70.5%)	23 (29.5%)		80 (72.1%)	31 (27.9%)	
Omentum	no	41 (85.4%)	7 (14.6%)	0.435 ^a^	58 (75.3%)	19 (24.7%)	0.596	99 (79.2%)	26 (20.8%)	0.848
	yes	11 (73.3%)	4 (26.7%)		35 (79.6%)	9 (20.4%)		46 (78.0%)	13 (22.0%)	
Concurrent injuries										
Soft tissue	no	39 (86.7%)	6 (13.3%)	0.173	84 (77.8%)	24 (22.2%)	0.604	123 (80.4%)	30 (19.6%)	0.280
	yes	13 (72.2%)	5 (27.8%)		13 (72.2%)	5 (27.8%)		26 (72.2%)	10 (27.8%)	
Ortho/neuro	no	33 (80.5%)	8 (19.5%)	0.733 ^a^	68 (77.3%)	20 (22.7%)	0.892	101 (78.3%)	28 (21.7%)	0.631
	yes	19 (86.4%)	3 (13.6%)		29 (78.4%)	8 (21.6%)		48 (81.4%)	11 (18.6%)	
Coexisting disease	no	41 (87.2%)	6 (12.8%)	0.093	81 (77.9%)	23 (22.1%)	0.602	122 (80.8%)	29 (19.2%)	0.189
	yes	11 (68.8%)	5 (31.2%)		16 (72.7%)	6 (27.3%)		27 (71.1%)	11 (28.9%)	
Surgery within 24 h	no	45 (83.3%)	9 (16.7%)	0.650 ^a^	86 (76.1%)	27 (23.9%)	0.731 ^a^	131 (78.4%)	36 (21.6%)	1.000 ^a^
	yes	7 (77.8%)	2 (22.2%)		11 (84.6%)	2 (15.4%)		18 (81.8%)	4 (18.2%)	
ASA score	2–3	30 (88.2%)	4 (11.8%)	0.319 ^a^	54 (77.1%)	16 (22.9%)	0.962	84 (80.8%)	20 (19.2%)	0.472
	4–5	22 (75.9%)	7 (24.1%)		43 (76.8%)	13 (23.2%)		65 (76.5%)	20 (23.5%)	
Total number of organs	0–2	34 (85.0%)	6 (15.0%)	0.497	44 (75.9%)	14 (24.1%)	0.879	78 (79.6%)	20 (20.4%)	0.717
	≥3	18 (78.3%)	5 (21.7%)		47 (77.1%)	14 (22.9%)		65 (77.4%)	19 (22.6%)	

PCV: packed cell volume, WBC: white blood cell, BUN: blood urea nitrogen, ALT: alanine aminotransferase, ASA: American Society of Anesthesiologists, * statistically significant at *p* < 0.05 (chi-squared analysis), ^a^ by Fisher’s exact test.

**Table 2:** Univariable logistic regression analysis of mortality in dogs and cats after diaphragmatic herniorrhaphy.

Variables		Odds Ratio (95% CI)	*p* Value
Species	Dog	Ref	0.379
	Cat	1.41 (0.65, 3.06)	
Sex	Female	Ref	0.279
	Male	0.68 (0.33, 1.37)	
Age	<5 years	Ref	0.161 *
	≥5 years	1.83 (0.79, 4.24)	
Weight (kg)	Dog <10/cat <3	Ref	0.662
	Dog ≥10/cat ≥3	1.17 (0.57, 2.40)	
Duration of hernia	Acute	Ref	0.003 *
	Chronic	3.01 (1.47, 6.18)	
Blood profiles			
PCV	Normal	Ref	0.270
	Anemia	0.65 (0.30, 1.40)	
WBC	Normal	Ref	0.051 *
	Leukocytosis	0.49 (0.24, 1.00)	
Plasma protein	Normal	Ref	0.686
	Abnormal	1.16 (0.56, 2.43)	
BUN	Normal	Ref	0.030 *
	Elevated	2.72 (1.10, 6.73)	
Creatinine	Normal	Ref	0.688
	Elevated	1.32 (0.33, 5.18)	
ALT	Normal	Ref	0.112 *
	Elevated	0.51 (0.22, 1.17)	
Anesthetics	Propofol	0.63 (0.23, 1.76)	0.381
	Alfaxalone	1.70 (0.61, 4.76)	0.311
	Isoflurane	0.99 (0.49, 2.00)	0.986
	Sevoflurane	1.00 (0.50, 2.02)	0.986
Surgical procedure	1 procedure	Ref	0.575
	>1 procedure	0.74 (0.26, 2.09)	
Herniated organ	Stomach	0.98 (0.48, 2.00)	0.959
	Liver	1.22 (0.53, 2.81)	0.630
	Spleen	0.79 (0.36, 1.73)	0.561
	Intestine	3.15 (1.35, 7.32)	0.008 *
	Omentum	1.08 (0.51, 2.28)	0.848
Concurrent injuries	Soft tissue	1.58 (0.69, 3.62)	0.283
	Ortho/neuro	0.83 (0.38, 1.80)	0.631
Coexisting disease	No	Ref	0.192 *
	Yes	1.71 (0.76, 3.85)	
Surgery within 24 h	No	Ref	0.716
	Yes	0.81 (0.26, 2.54)	
ASA score	2–3	Ref	0.472
	4–5	1.29 (0.64, 2.60)	
Total number of organs	0–2	Ref	0.717
	≥3	1.14 (0.56, 2.32)	

CI: confidence interval, Ref: reference, PCV: packed cell volume, WBC: white blood cell, BUN: blood urea nitrogen, ALT: alanine aminotransferase, ASA: American Society of Anesthesiologists, * *p* value < 0.2.

**Table 3:** Final multivariable logistic regression model with mortality as the outcome variable.

Variables		Adjusted Odds Ratio (95%CI)	*p* Value
Age	<5 years	Ref	0.1707
	≥5 years	1.91 (0.76, 4.85)	
Duration of hernia	Acute	Ref	0.0016 *
	Chronic	4.01 (1.69, 9.53)	
BUN level	Normal	Ref	0.0181 *
	Elevated	3.24 (1.22, 8.57)	

CI: confidence interval, Ref: reference, BUN: blood urea nitrogen. * Statistically significant at *p* < 0.05.

**Table 4:** Multivariable Cox proportional hazards model for one-year survival in dogs and cats with diaphragmatic hernia. The table presents hazard ratios (HRs) with 95% confidence intervals (CIs) and *p*-values for each variable.

Variables		Adjusted Hazard Ratio (95%CI)	*p* Value
Age	<5 years	Ref	0.367
	≥5 years	1.49 (0.63, 3.55)	
Duration of hernia	Acute	Ref	0.003 *
	Chronic	3.31 (1.51, 7.30)	
BUN level	Normal	Ref	0.015 *
	Elevated	2.88 (1.23, 6.77)	

CI: confidence interval, Ref: reference, BUN: blood urea nitrogen. * Statistically significant at *p* < 0.05.

## Data Availability

The datasets used and/or analyzed during the current study are available from the corresponding author on reasonable request.

## Abbreviations

The following abbreviations are used in this manuscript.

DH	diaphragmatic hernia
NCSS	Number Cruncher Statistical System
ROC	receiver operating characteristic
AUC	area under the ROC curve
OR	odds ratio
CI	confidence interval
BUN	blood urea nitrogen
CART	classification and regression tree
ASA	American Society of Anesthesiologists
PCV	packed cell volume
WBC	white blood cell
ALT	alanine aminotransferase
Ref	reference
OOB	out of bag

## Appendix A

**Table A1:** Reference ranges of hematology and blood chemistry in dogs and cats.

Parameters	Canine	Feline
PCV (%)	35–57	30–45
WBC count (×10^3^/µL)	5.0–14.1	5.5–19.5
Platelet count (×10^3^/µL)	211–621	300–800
BUN (mg/dL)	8–28	19–34
Creatinine (mg/dL)	0.5–1.7	0.9–2.2
ALT (U/L)	10–109	25–97
Plasma protein (Biuret) (g/dL)	5.4–7.5	6.0–7.9
Albumin (g/dL)	2.3–3.1	2.8–3.9

Reference intervals issued by the Veterinary Diagnostic Laboratory of the Faculty of Veterinary Medicine at Kasetsart University (as of 14 August 2020 update) based on veterinary laboratory guidelines [47].
